# Oxidative Stress, Inflammation, and Antioxidant Strategies in Cervical Cancer—A Narrative Review

**DOI:** 10.3390/ijms26104961

**Published:** 2025-05-21

**Authors:** Ecaterina Tomaziu-Todosia Anton, Gabriel-Ioan Anton, Ioana-Sadiye Scripcariu, Irina Dumitrașcu, Dragos Viorel Scripcariu, Ioana-Miruna Balmus, Cătălina Ionescu, Mălina Visternicu, Demetra Gabriela Socolov

**Affiliations:** 1 Department of Obstetrics and Gynecology, Faculty of Medicine, “Grigore T. Popa” University of Medicine and Pharmacy, University Street, No. 16, 700115 Iasi, Romania; 2 Department of Obstetrics and Gynecology, Clinical Hospital of Obstetrics and Gynecology “Cuza Voda”, Cuza Voda Street, No. 34, 700038 Iasi, Romania; 3 Department of Surgical Specialties I, Faculty of Medicine, “Grigore T. Popa” University of Medicine and Pharmacy, University Street, No. 16, 700115 Iasi, Romania; 4 Department of Surgery, Regional Institute of Oncology Iasi, General Henri Mathias Berthelot Street, No. 2-4, 700483 Iasi, Romania; 5 Department of Exact Sciences and Natural Sciences, Institute of Interdisciplinary Research, “Alexandru Ioan Cuza” University of Iasi, Alexandru Lapusneanu Street, No. 26, 700506 Iasi, Romania; 6 CENEMED Platform for Interdisciplinary Research, “Grigore T. Popa” University of Medicine and Pharmacy, University Street, No. 16, 700115 Iasi, Romania; 7 Department of Biology, Faculty of Biology, “Alexandru Ioan Cuza” University of Iași, Carol I Avenue, 20A, 700506 Iasi, Romania; 8 Doctoral School of Biology, Faculty of Biology, “Alexandru Ioan Cuza” University of Iași, Carol I Avenue, 20A, 700506 Iasi, Romania

**Keywords:** cervical cancer, cervical intraepithelial neoplasia, HPV, oxidative stress, inflammation

## Abstract

Cervical cancer ranks third among malignant diseases of the female reproductive system and progressively develops through a series of pathological changes known as cervical intraepithelial neoplasia (CIN). Despite being extremely aggressive and causing increased mortality, the main treatment options include surgery or a combination of chemotherapy and radiotherapy, often based on cisplatin-based chemotherapy and external beam radiotherapy or brachytherapy. Cervical dysplasia is an abnormal growth of cells on the surface of the cervix that could lead to cervical cancer. CIN most commonly occurs at the squamocolumnar junction of the cervix, a transitional zone between the squamous epithelium of the vagina and the columnar epithelium of the endocervix. The primary cause of CIN is chronic infection of the cervix with Human Papillomavirus (HPV). Oxidative stress (OS) and chronic inflammation are associated with HPV-induced cervical dysplasia. Reactive oxygen species (ROS) facilitate the progression of CIN through DNA damage, immune evasion, and cellular mutations. Thus, the inflammatory environment, characterized by increased expression of proinflammatory cytokines, contributes to epithelial transformation. Given these mechanisms, antioxidants, including vitamins A, C, D, E, polyphenols, and carotenoids, are being investigated for their potential as adjunctive therapies in CIN management. This review aims to provide a comprehensive analysis of the influence of oxidative stress, antioxidants, and inflammation on cervical cancer.

## 1. Introduction

Cervical cancer is the fourth most common malignancy in women worldwide, following breast and colorectal cancer [1]. Cervical cancer develops through a multi-step process involving the transformation of epithelial cells due to persistent Human Papillomavirus (HPV) infection, especially the strains with oncogenic potential, leading to a precancerous condition known as cervical intraepithelial neoplasia [2]. The incidence of CIN is higher in less developed countries due to limited screening and the high cost of the HPV vaccine. Risk factors for cervical cancer include both behavioral and infectious factors [3]. These include age at first sexual intercourse, number of sexual partners, parity, smoking, co-infections, long-term use of oral contraception, and cervical dysplasia [4]. Behavioral factors, such as sexual activity and lifestyle, also play an important role in increasing the risk of developing CIN [3].

Being the most common premalignant lesion of the cervix, CIN is commonly characterized by atypical squamous changes occurring at the junction between the endocervix and ectocervix (cervical transformation zone). The diagnosis is performed by histopathological methods, while the classification depends on the depth of the lesions [5,6]. The mechanism through which the increased oncogenic strains of HPV could cause CIN and eventually cervical cancer includes a complex interaction between virulence, viral multiplication, and viral proteins in cellular dysplastic changes [7].

Concurrent non-specific mechanisms, such as oxidative stress (OS) and inflammatory responses, may also contribute to HPV infection persistence, CIN pathogenesis, and malignant processes. Reactive oxygen species (ROS) that are commonly produced during OS were previously shown to contribute to lipid peroxidation and DNA damage, both processes being associated with malignant mechanisms, including cervical cancer [8,9,10]. In this context, many studies have suggested that antioxidant defense could play an important role in modulating the neoplastic transformations and HPV-persistent infections [8,11,12]. Also, dietary-associated nutrient deficits, such as hypovitaminoses and microelement insufficiencies, were suggested as main contributors to OS and DNA damage [11]. Furthermore, the connection between OS and inflammation was known far before the connection between OS and malignant processes. The contribution of immune factors, including cytokines and infiltrating cells, has been identified in cancer development. Additionally, strong correlations between DNA damage or ROS-associated extracellular matrix disruption and the stimulation of pro-inflammatory cytokine activity were shown in tumor growth processes [13]. In this context, previous studies have shown that infiltrating leukocytes and uterine epithelial cells are locally expressing various cytokines that modulate the occurrence of CIN and further transformation to cervical cancer by being involved in angiogenesis, apoptosis, and chemotaxis, processes that can modulate the progression of dysplasia [13].

However, a thorough description of the interaction between these three players has only been scarcely performed in the context of CIN and cervical cancer. Thus, the current study aims to describe the influence of OS on inflammatory processes contributing to CIN and cervical cancer, by providing a comprehensive analysis of the pathophysiological mechanisms and potential therapeutic strategies.

## 2. Cervical Intraepithelial Neoplasia

Cervical intraepithelial neoplasia is a pathogenic condition closely related to persistent HPV infection, characterized by the abnormal transformation of epithelial cells comprising the cervical mucosal layers [14]. While left untreated or not discovered in incipient stages, HPV-induced CIN precedes the development of cervical cancer, usually the cervical squamous cell carcinoma [15]. It was recently shown that the natural progression of CIN could be due to an interaction between HPV virulence and multiplication, host factors, and environmental factors [16]. Most commonly associated with CIN development are the lifestyle factors, such as obesity, smoking, contraceptive use, and the presence of vaginal infections [17].

Despite the recent studies that have shown multiple causes of CIN, the most prevalent cause remains HPV infections [14]. The major association between cervical cancer and HPV infection makes the latter a major challenge for global health, being one of the leading causes of female mortality worldwide [18]. In all regions, the highest prevalence of HPV has been reported in women under 35 years of age, who are generally sexually active, suggesting a main sexual infection route [19]. It is important to note that not all HPV strains are associated with cervical cancer development. Studies have described 18 cancer-associated types of HPV [20]. HPV infection could occur via skin-to-skin contact, skin-to-mucosa contact, horizontal transmission (via fomites), autoinoculation, vertical transmission (from mother to newborn), and sexual transmission, suggesting a very efficient mechanism of virulence [21]. Thus, CIN pathogenesis debuts when HPV penetrates the cervical epithelium through micro-abrasions upon contact, particularly HPV-16 and HPV-18 strains [22]. While rapidly reaching the basal layer of the epithelia, the integration of the viral genetic material into the host cell’s DNA is initiated, promoting persistent infection—a crucial factor of progression to CIN [23]. In this process, the viral oncoproteins E6 and E7 play a central role, as E6 induces the degradation of the p53 protein and prevents apoptosis of the infected cells, while E7 inhibits the tumor-suppressor protein retinoblastoma, promoting uncontrolled cell division [24]. All these changes lead to excessive proliferation of the infected epithelial cells, evading natural regulatory mechanisms. The prolonged expression of these oncogenes may cause genetic mutations and chromosomal instability, promoting the progression of precancerous lesions and their evolution into CIN [25,26]. However, the true challenge resides in understanding the nature of the interaction between the genome of HPV and the immune system of the host that leads to the development of cervical cancer [27]. While women previously infected with HPV are more likely to acquire two or more subtypes, it is still not clear how the viral loads affect the development of precancerous lesions but are not associated with the severity of the lesions [20,28].

Increasing evidence vouches for the significant risk of developing CIN in tobacco users [29,30,31]. High-grade CIN and invasive carcinoma were reported twice as frequently in tobacco users, especially when consumed as cigars, while both time and frequency of smoking were significant contributing factors, in addition to HPV infection [32]. Awareness should be raised regarding the potential of cigar smoke to contribute to cervical carcinogenesis, even in passive exposure conditions [33,34].

The endocrine modulation of CIN risk was also widely debated. In this way, several studies have shown that estrogen could be actively involved in both the predisposition and development of CIN [35]. A recent systematic review evaluating the relationship between hormonal contraception and HPV-related CIN reported that no consistent evidence could demonstrate this issue [36]. However, the reports on this correlation are often contradictory. Other studies described the direct association between long-term oral contraceptive use and the increased risk for cervical cancer, as compared to non-users [37]. Additionally, they reported that the risk decreased 10 years after discontinuing oral contraceptives, suggesting that estrogen levels are involved. Moreover, Wu et al. [34] also explained that estrogen and progesterone could be implicated in HPV replication by promoting E6 and E7 oncoprotein expression.

Interdisciplinary approaches are required for the classification and management of lesions. The initial screening comprises cytological analyses, while imaging techniques, such as colposcopy, are used to live evaluate the cervical mucosa and guide bioptic maneuvers. The gold standard for CIN diagnosis is histological evaluations, such as Pap smears [14,38].

The stabilization of CIN is currently achieved by the original system developed by Richart [39], depending on the severity of the lesions: CIN1—low grade lesions, CIN2, CIN3—moderate to severe lesions. The traditional grading of CIN is based on the proportion of epithelial thickness affected by undifferentiated basal cells [14,40,41] (Table 1). The current system of CIN stabilization could also offer valuable information regarding the predisposition of the confirmed lesions to progress to cervical cancer [41].

Current management approaches of CIN include both monitoring strategies and surgical interventions. Watchful waiting approaches are commonly recommended for CIN1 cases in which the lesions have an increased potential of spontaneous regression, especially in young women. Meanwhile, patients are monitored by screening tests, including Pap smears and HPV testing, to track lesion progression [44,45]. Recent studies have, however, shown that watchful waiting could not be as efficient as previously thought and innovative treatments for low-grade cervical lesions have emerged, such as the fungus-based vaginal gel. In this way, several studies [46,47,48] have reported important benefits of *Coriolus versicolor* extract in treating HPV-originating cervical lesions.

The initial observational management of CIN2 is increasingly accepted as appropriate for women under 25 years of age with abnormalities detected during screening and is included in a series of clinical guidelines [49]. Since cytological and histological screening cannot detect the potential of CIN2 lesions to develop into cervical cancer, it is essential to develop specific biomarkers that enable the clinical differentiation between women with CIN2 who require immediate excision and those who only need careful long-term monitoring [16]. On the other hand, CIN3 showed increased progression and decreased regression potentials [38]. Generally, CIN3 development is caused by the infection with highly oncogenic HPV strains (HPV-16 and HPV-18) [50]. The initiation of cervical dysplasia is modulated by the normal homeostasis disruption of the cervical epithelium as a result of the viral localization in the basal epithelial cells of the cervical transformation zone [51]. In this way, the expression of several viral oncoproteins, particularly E6 and E7, modulates key cellular regulatory mechanisms that control apoptosis and tumor suppression [22].

Nevertheless, some cases of CIN2 and most of CIN3 carry an increased potential in progression to invasive cervical cancer and may require more active treatments. In this case, the clinical guidelines of monitoring include more frequent tests. While the lesions are persistent or aggravating, interventional treatments may be considered. The loop electrosurgical excision (LEEP) is a minimally invasive procedure that uses a wire loop electrode to excise abnormal cervical tissue and instantly cauterize the immediate surroundings, including precancerous lesions and the entire transformation zone of the cervix [52,53,54]. Several complications of the LEEP procedure have been reported with minimum life-threatening risk: intraoperative bleeding (3.4%), infections (4.9%), and postoperative bleeding (5.3%) [52]. By comparison to LEEP, cold knife conization is a more invasive surgical technique which is used for moderate lesions. This procedure involves removing a cone-shaped portion of the cervical tissue for both treatment and bioptic reasons [2,55]. More severe lesions are surgically treated by laser therapy involving the administration of a thin controlled laser beam to remove abnormal tissue. The latter surgical intervention exhibits net advantages over the others while being characterized by short recovery time and minimal blood loss [56].

For CIN cases with lesser span and severity, minimally invasive techniques are preferred and have also been described. Cryotherapy is an effective advantageous procedure for treating CIN1 and early-stage CIN2 lesions and is based on the instant destructive effect of extreme cold over the abnormal tissues [57]. Additionally, cryotherapy implicates minimal health-threatening risks, with significantly fewer major bleedings and infections, as compared to cold knife conization and LEEP [52,58]. By contrast to cold therapy but with similar principles, thermal ablation uses controlled heat to destroy abnormal tissue.

Recently, immunotherapy has been reported as a promising approach in CIN treatment, by using therapeutic vaccines to stimulate the immune system in targeting HPV-associated abnormal cells [59]. Most of these vaccines target HPV E6 and E7 proteins and are currently tested in preclinical and clinical studies [60]. Peptide- and protein-based vaccines use HPV proteins’ fragments and adjuvants to stimulate immune responses [61]. The immunogenic effects in cervical cancer patients were characterized by the stimulation of specific responses against HPV and a positive impact on the regression of precancerous lesions and HPV-associated cancers [62]. By contrast, live vector vaccines stimulate immune responses through antigen processing in macrophages with positive effects in 40% of cervical cancer patients [63]. On the other hand, both cellular and humoral responses against HPV could be induced by nucleic acid-based vaccines [60]. Cell-based vaccines stimulate the reversal of HPV-related diseases by extracting and modifying cells to produce cytokines, which are then injected to induce infection regression, yet further studies are needed to evaluate their long-term efficacy and safety associated with the reported immunogenic effects [60]. However, it is worth mentioning that preventive methods, such as HPV vaccination and periodic cervical screening, are preferred over the treatment methods [64]. The introduction of systematic screening programs has led to a profound decrease in the cervical cancer incidence and mortality [41,65].

## 3. Oxidative Stress and Inflammation in the Pathogenesis of CIN

ROS are a category of highly reactive molecules, primarily consisting of free radicals—atoms or molecules with unpaired electrons that drive their reactivity. While free radicals can be derived from various elements, oxygen- and nitrogen-based species are the most dominant in human homeostasis [66]. Under normal conditions, ROS are continuously produced in small quantities within cellular compartments, such as the mitochondrial respiratory chain [67] and the endoplasmic reticulum [68]. ROS and RNS are mainly second messengers in cell signaling pathways. Through the activation of protein kinases, regulation of ion channels, modulation of transcription factors, and involvement in apoptosis and protein modifications, ROS contribute to regulation of cellular processes [69].

In this way, as cells are constantly exposed to various internal and external factors, including OS, DNA and RNA damage could occur. Genetic material damage could lead to replication and transcription impairment, apoptosis, as well as mutations and neoplastic transformation [70]. The correlations between ROS excessive production and subsequent damaging effects were shown in degenerative conditions, including neurological disorders [71], cardiac dysfunction [72], cancer [73], and even during the natural aging process [74].

Although ROS are often associated with cellular damage, they also act as signaling molecules in response to stress. Recent studies suggested that DNA damage itself can lead to increased intracellular ROS levels. DNA double-strand breaks were reported to trigger mutations, which could activate oncogenes, deactivate tumor suppressor genes, and modify the activity or expression of proteins involved in chemosensitivity and tumor progression [75]. The natural response to DNA damage is a complex mechanism that detects DNA damage, halts the cell cycle allowing the repair, or activates programmed cell death [75]. Studies on the cellular DNA repair mechanisms were performed in Saccharomyces cerevisiae and reported that the counteraction of OS is promoted by the activation of several transcription factors regulating the expression of enzymes responsible for ROS detoxification and metabolism [76].

While a balance between the formation and the neutralization of ROS is generally assisted by the antioxidant defense system that is active in all the body cells, the loss of this balance could lead to OS [77]. This imbalance between oxidants and antioxidants has been studied in numerous cancers, including cervical cancer. Key factors contributing to the development of cervical cancer include chronic inflammation caused by cervical trauma, bacterial infections, and, most notably, HPV [78]. Consequently, extensive evidence showed that HPV infection could contribute to OS in cervical cancer development [9]. Also, persistent inflammation triggers the activation of the monocyte/macrophage system, leading to excessive production of ROS, sustained OS, and excessive inflammatory response [79]. Additionally, ROS generation induces genetic alterations in the cervical epithelium, facilitating malignant transformation and the onset of cervical cancer [9].

Despite the use of standard diagnosis and treatment protocols (PAP smear, HPV genotyping, colposcopy, biopsy, radical surgery, chemotherapy, and radiotherapy), cervical cancer still has a high incidence and mortality rate worldwide [80]. It was previously extensively shown that OS plays an essential role in the initiation and progression of cancer, due to ROS metabolism imbalance [81,82]. Increased levels of ROS have been detected in many malignant processes, being a well-characterized feature of cancer phenotypes [9]. Previous reports have demonstrated that moderate ROS levels could stimulate cellular stress signaling and contribute to genetic material mutations [83]. Furthermore, OS-associated chronic inflammation was shown to promote various types of cellular damage, including malignant processes [80]. Also, ROS-dependent tumorigenesis was associated with mitochondrial activation of redox signaling [81]. During mitochondrial activation, cytochrome P450 and peroxisomes contribute as major endogenous promoters of ROS and reactive nitrogen species (RNS) synthesis. Additionally, ROS production could also be modulated by exogenous factors, such as radiation, smoking, chemotherapy, and diet, some of which were already documented as independent predisposition factors for CIN development [82].

In cervical cancer, OS was highlighted by a significant increase in OS-specific biomarkers and attributed to the dysregulation of antioxidant defense mechanisms [79]. Manju et al. [83] evaluated the implication of OS in a group of Indian patients diagnosed with moderate to severe cervical cancer. As compared to a group of healthy age-matched control individuals, the patients exhibited significant decreases of in glutathione (GSH) and enzymes such as glutathione S-transferase (GST), glutathione peroxidase (GPx), and superoxide dismutase (SOD) antioxidant blood levels. Additionally, some of the plasmatic non-enzymatic antioxidant levels (vitamins C and E) were also significantly decreased in cervical cancer patients, suggesting that redox imbalance in cervical cancer could be the result of antioxidant system impairment [84]. Similar patterns of variation were seen for SOD and GPx activity in other studies (Table 2).

However, in some cases, the decrease in the antioxidant defense barrier could not be associated with cellular damage, as it could be suggested by malondialdehyde (MDA) level changes [85]. By contrast, a case-control institutional study recently reported significant increases in MDA and SOD levels and GSH levels decrease in cervical cancer patients’ serum, as compared to healthy age-matched controls [9]. Additionally, they reported increased levels of 8-OHdG in patients’ serum, suggesting that OS could generate RNA lesions (especially messenger RNA and ribosomal RNA) that are tied to defective translational processes and impairment of protein synthesis [86]. OS-associated DNA damage was reported by Naidu et al. [87] as they observed significant increases of MDA and nitric oxide (NO) levels in the blood of patients who were diagnosed with cervical cancer, as compared to healthy age-matched controls. Additionally, they suggested that the decreased levels of vitamin C and SOD in the red blood cells of the patients may contribute to oxidative damage potentiation. Thus, taken together, these changes could lead to a significant negative impact on cellular integrity, while vitamin C hypovitaminosis contributes to increasing the effects of OS on the body [86,87].

It was previously shown that, in malignant cells, OS is a consequence of oncogenic mutations that lead to altered cellular metabolism. One of the main contributors to elevated OS in cancer is the excessive production of ROS [88]. With ROS being potent secondary messengers in intracellular signaling, their excessive synthesis and accumulation contribute to the promotion of cancer cells’ survival. Interestingly, cancer cells show a higher resistance to OS than normal cells. Particularly, it was shown that HPV-infected cells could adapt to OS by enhancing the activity of protective enzymes, such as SOD and CAT [89]. Thus, cancer cells primarily manage OS-induced damage by regulating antioxidant activity and inhibiting apoptosis triggered by oxidative stress [89,90].

Although not yet conclusively proven, it is believed that the early stages of HPV infection—such as viral adsorption, entry, and initial viral expression—play a crucial role in determining the outcome of the infection, which could range from spontaneous healing to productive and persistent infection, or even neoplastic transformation. Clinical and epidemiological observations have provided evidence of OS involvement during these phases. For instance, a study on cervical cancer investigating diamine and polyamine levels in seminal plasma, as well as oxidative enzymes in cervical mucus, suggested that variations in local oxidative stress could influence the progression of the infection [91]. Another study on 327 serum samples found that women with high ferritin levels were less likely to clear high-risk HPV infections, possibly due to increased ROS generation [92]. Additionally, a large genetic study linked a polymorphism of the gene encoding an important antioxidant enzyme found in the mitochondria (PRDX3) to higher infection persistence and increased progression risk [93]. Recent data from organotypic cultures demonstrated that the complete replication the high-risk HPV-16 strain depends on a tissue-spanning redox gradient, indicating that redox balance affects viral titer and may contribute to infection persistence [94].

Furthermore, it was shown that HPV oncoproteins could disrupt mitochondrial function, generating OS, DNA damage, and subsequent carcinogenesis. Lai et al. [95] and Cruz-Gregorio et al. [96] were just a few who have documented the implication of HPV-16 E1/E4 and HPV-18 E2 in mitochondrial structure alteration by boosting ROS production and shifting the metabolism towards glycolysis (the process known as the Warburg effect). Moreover, HPV-16/18 E2 and E1, respectively, were shown to amplify ROS and DNA damage, while HPV-16 E2 was shown to trigger apoptosis via the p38 MAPK/JNK pathway [95,96]. E6/E7 oncoproteins were reported as potent impairers of mitochondrial RNA expression [97,98]. In some cases, despite increasing mitochondrial activity, HPV oncoproteins could promote respiration leakage rather than ATP production, leading to more ROS and DNA damage. In this way, HPV-16 E7 could reduce OS by upregulating CAT, preventing apoptosis in keratinocytes, while in HPV+ head and neck cancer, E6/E7 induce OS through NOX enzymes, causing DNA instability [99].

As HPV infection triggers inflammation through NF-κB activation, OS is often accompanied by cytokine production and immune cell recruitment. Chronic inflammation, driven by persistent infection, increases ROS, DNA damage, and carcinogenesis risk [100,101]. It was shown that HPV could evade immune responses by downregulating key immune components, promoting viral persistence, malignancy progression, and pro-inflammatory microenvironment via IL-1β, IL-6, and immune cell recruitment [102,103]. In addition, HPV could regulate inflammatory chemokines (such as CXCL1-3, CXCL8), while OS contributes to viral DNA integration by promoting genome instability and cancer development. Significant evidence correlated high-risk HPV strains with increased cytokines levels, lipid peroxidation, and oxidative DNA damage [104,105,106].

While the correlation between OS and inflammation was previously extensively studied in many types of malignant processes, it was shown that chronic inflammation plays a crucial role in cancer development and is implicated in various epithelial cancers, including stomach, colorectal, and bladder cancer. Inflammatory response could act as a cancer promoter by inducing cell proliferation, recruiting inflammatory cells, increasing oxidative DNA damage, and impairing DNA repair, all contributing to carcinogenesis [107]. The activation of immune cells (neutrophils, eosinophils, monocytes, mast cells, platelets, and fibroblasts) that migrate to the site of injury or infection modulate the release of many cytokines, ROS, and hormones, to sustain the inflammatory response. Although infectious and autoimmune mechanisms are the main causes of prolonged inflammation, in many cases, the exact cause remains unknown. However, chronic inflammation plays a significant role in cancer development by promoting cellular transformation, proliferation, invasion, and angiogenesis [90]. Inflammatory cytokines play a major role in tumor development by disrupting essential signaling pathways. While the innate immune system uses TLR receptors for defense, their excessive activation can lead to chronic inflammation and tumor promotion. Mutated TNF-α can directly contribute to cancer formation, while cytokines such as IL-6, IL-8, and IL-1β stimulate cell proliferation and cervical cancer progression [108].

Additionally, signaling pathways like NF-κB and JAK/STAT are crucial in carcinogenesis, aiding immune evasion and creating an immunosuppressive tumor environment. The HPV oncogenic proteins E6/E7 promote STAT3 expression and inhibit PRb and p53, facilitating cancer progression. Dysregulation of NF-κB leads to persistent inflammation, abnormal proliferation, and metastasis, highlighting the interconnected nature of these pathways in cancer development [109]. Other inflammatory mechanisms that promote tumor growth include DNA damage and extracellular matrix degradation by ROS and metalloproteinases [13]. Cytokines, such as IL-1β and IL-8, stimulate tumor proliferation. The immune system, through inflammation, alters both tumor cells and their microenvironment. Monocytes and macrophages, the main tumor-infiltrating cells, produce cytokines like IL-1β, IL-6, IL-23, and TNF-α, playing a crucial role in cancer progression within an inflammatory context [13]. Furthermore, a correlation between inflammation, angiogenesis, and cancer was described. While it was reported that 16% of cancers could be caused by infections, more than one-fourth of all inflammatory processes are believed to contribute to tumor development [109].

Unlike acute inflammation, chronic inflammation involves inflammatory infiltrates primarily composed of mononuclear cells that produce ROS and RNS that contribute to microbe clearance but also to cell and DNA damage leading to carcinogenesis. In this case, some pro-inflammatory cytokines (IL-1 and IL-6) and some growth factors (TGF-β and VEGF) could activate NF-κB and STAT3 signaling pathways to promote the survival of damaged cells. Other inflammatory factors, including VEGF-A, cyclooxygenase-2, and prostaglandins, influence the endothelium, enhancing cell recruitment, inflammatory mediator production, vascular permeability, and angiogenesis, all of which are crucial for tumor progression [109].

## 4. Antioxidants in the Pathogenesis and Treatment of CIN

Briefly, in cervical cancer, OS results from imbalanced antioxidant defense mechanisms. Antioxidants, found in plasma, red blood cells, and tissues, are categorized as enzymatic (e.g., SOD, CAT, GPx) and non-enzymatic (e.g., GSH, vitamins C, E, A). Enzymatic antioxidants serve as the first line of defense by neutralizing ROS, while secondary enzymes like GST and glutathione reductase (GR) aid in detoxification. Non-enzymatic antioxidants support both detoxification and repair of oxidative damage. A better understanding of these defense systems could help improve cervical cancer prognosis and treatment strategies [79]. In cancer treatment, chemotherapeutic agents generate free radicals to damage malignant cells, with extensive side effects on the redox homeostasis. Similarly, radiotherapy, while effective, can induce DNA. Certain antioxidant supplements may help reduce these side effects by neutralizing oxidative damage and protecting DNA from cancer initiation [110,111].

In this way, recent studies confirmed that many natural compounds possess chemotherapeutic and chemopreventive properties partly due to antioxidant characteristics. In this context, an inverse association has been observed between dietary antioxidant factors and the development of HPV-induced tumors, suggesting that natural antioxidants play a protective role against HPV persistence and tumor development [82]. The main sources of natural antioxidants include fruits, vegetables, whole grains, green and black tea, beer, wine, coffee, and spices [81]. Recently, many plant-derived polyphenols have drawn attention as effective anticancer agents due to their ability to act on multiple targets and their reduced side effects [112].

Quercetin is consumed daily by millions of people through nuts, tea, vegetables, and is also available as a commercial dietary supplement. Approximately 60% of orally ingested quercetin is absorbed, and the average daily intake from food varies between 6 and 31 mg, excluding supplements or intravenous use [113]. Abundant evidence was reported regarding the effects of quercetin on HeLa cervical cancer cells (Table 3) [114,115,116]. In one study, researchers investigated the effects of quercetin on HeLa cells at concentrations of 20, 40, and 80 μmol/L for 24, 48, and 72 h. It was found that the viability of HeLa cells was significantly inhibited in the quercetin-treated group, suggesting a strong tumor growth inhibitory effect [117]. Similarly, in another study, researchers administered 25 and 50 μM quercetin for 24 and 48 h to HeLa cell cultures and obtained cell proliferation inhibition via accumulation of cells in the G2/M phase of the cell cycle. Moreover, quercetin induced DNA fragmentation and apoptosis through the modulation of the PI3K, MAPK, and WNT signaling pathways, further supporting its potential as a strong anticancer agent [115]. Xu et al. [116] showed that quercetin (0–200 μM, 24–48 h) enhances the cytotoxic effects of cisplatin in HeLa cells, stimulating apoptosis and inhibiting proliferation, migration, and invasion. These results could suggest quercetin as a potential adjuvant in cervical cancer chemotherapy [116]. Furthermore, Priyadarsini et al. [114] reported that quercetin inhibited HeLa cell proliferation at concentrations of 20–100 μM for 24 h by blocking the cell cycle at the G2/M phase and inducing apoptosis, affecting the p53 and NF-κB pathways involved in cancer progression.

In addition to quercetin, other natural compounds, such as resveratrol, curcumin, rosmarinic acid, 6-gingerol, and kaempferol have shown promising effects on HeLa cells [118,119,120,121,122,123]. Liu et al. [116] found that resveratrol (0, 40, and 80 μmol/L) inhibited cell proliferation and induced apoptosis by activating FOXO3a nuclear translocation. Resveratrol is a representative phytoalexin of dietary-derived chemopreventive cancer agents, which has attracted the attention of the cancer chemoprevention community. This natural compound has a variety of biological activities, including anti-inflammatory, antioxidant, immunoregulatory, chemopreventive, and antitumor effects [122]. It has been found to have antiproliferative effects on cervical cancer cell lines [82]. Moreover, it has been shown to inhibit the action of HPV E6 and E7, induce apoptosis, and reduce the viability of cancer cells, including human cervical cancer cells [122].

**Table 3:** Effects of antioxidants on cervical cancer.

Antioxidant	Model/Patients	Study	Doses	Treatment Duration	Effect	Ref.
In vitro experimental models						
Quercetin	HeLa cell culture	Exploring the effect of quercetin in cervical cancer cells obtained by endoplasmic reticulum stress and apoptosis-modulated tumor induction.	20, 40, and 80 μmol/L	24 h, 48 h, and 72 h	The viability of HeLa cells was significantly inhibited in the quercetin-treated group.	[117]
	HeLa cell culture	Investigating the effects of quercetin on apoptosis, proliferation, and tumorigenesis.	25 and 50 μM	24 and 48 h	Quercetin inhibits cell proliferation halting the cell cycle, causes DNA damage, and induces apoptosis in HeLa cells by modifying the PI3K, MAPK, and WNT pathways.	[115]
	HeLa cell culture	Examining the impact of quercetin on the efficacy of various chemotherapeutic drugs in cervical cancer cells.	0, 10, 20, 50, 100, 150, and 200 μM	24 h and 48 h, respectively	Quercetin enhanced the cytotoxic effects of cisplatin, promoting apoptosis and inhibiting proliferation, migration, and invasion of cervical cancer cells.	[116]
	HeLa cell culture	Examining the effects of quercetin on cell viability and its mechanism of inducing cell death by analyzing the expression of key proteins involved in apoptosis, cell cycle regulation, and NF-κB signaling in HeLa cells.	20, 40, 60, 80, and 100 μM	24 h	Quercetin inhibits the proliferation of HeLa cells by arresting the cell cycle at the G2/M phase and inducing apoptosis. It also simultaneously targets two opposing signaling pathways—p53 and NF-κB—to inhibit cancer progression.	[114]
Resveratrol	HeLa cell culture	Exploring how resveratrol, as an anticancer agent, effectively influences HeLa cervical cancer cells.	0, 40, 80 μmol/L	48 h	Resveratrol inhibited cell proliferation and induced apoptosis. Mechanistically, resveratrol facilitated the nuclear translocation of the transcription factor FOXO3a, leading to increased expression of the pro-apoptotic protein BIM.	[118]
Curcumin	HeLa cell culture	Exploring the effect of curcumin administration on p53 and caspase-3 in HeLa cell line.	25, 50, 100, 150, and 250 µg/mL	24 h	Curcumin treatment significantly promoted apoptosis and upregulated p53 and caspase-3 expressions. At a concentration of 100 µg/mL, total apoptosis significantly increased.	[121]
Rosmarinic acid	HeLa cell culture	Examining the biological properties of rosmarinic acid from the methanolic extract of *Mentha piperita* L., focusing on its cytotoxic effects on breast cancer, HeLa cell lines, and normal cells in vitro.	100, 250, 500, and 1000 μg/mL.	48 h	Rosmarinic acid inhibited MCF-7 breast cancer cells by 48% at 1000 µg/mL, while HeLa cervical cancer cells showed only 1% inhibition, but it did not investigate the effects of rosemary oil.	[120]
6-Gingerol	HeLa cell culture	Observing the inhibitory effect of 6-gingerol on the invasion and migration of both HPV-positive and HPV-negative cervical cancer cells and investigating the underlying mechanism.	0, 5, 10, 20, and 50 μmol/L	24 h	Activity of HeLa cells decreased with the increase of 6-gingerol concentration.	[123]
Kaempferol	HeLa cell culture	Investigating the anti-proliferative effect of kaempferol on HeLa and AC 16 cells and identifying the molecular targets involved in its anticancer properties.	30, 40, and 50 μM	48 h	Kaempferol induced apoptosis, disrupted mitochondrial potential, and led to the accumulation of cells in the G2-M phase of the cell cycle.	[119]
Animal models						
*Curcuma amada* rhizome extract	30 female Sprague Dawley rats	Evaluating the antioxidant and chemotherapeutic potential of *Curcuma amada* rhizome extract on benzo(α)pyrene-induced cervical carcinoma in rats.	Oral (250 mg and 500 mg ethanol extract)	8 weeks BaP exposure + 4 weeks post-treatment	Reducing tumor burden, restoring antioxidant levels, decreasing lipid peroxidation, and improving membrane-bound enzyme activities.	[124]
Vitamin E	25 Swiss albino female mice	Investigating the effect of vitamin E supplementation on cervical epithelial carcinogenesis.	Gavage (100 mg/kg body weight/day)	30 days carcinogen exposure with concurrent treatment	Reducing the incidence of dysplasia, preventing carcinoma in situ, improving antioxidant enzyme activity, decreasing lipid peroxidation, and supporting the immune response.	[125]
Curcumin and beta-carotene	56 female Wistar albino rats	Evaluation of the potential protective effects of curcumin and beta-carotene against cisplatin-induced ovarian damage.	Curcumin: 200 mg/kg, beta-carotene: 100 mg/kg, cisplatin: 5 mg/kg	1 week	Curcumin and beta-carotene protected against cisplatin-induced follicle loss and reduced NF-kB levels. Curcumin has more antioxidant and anti-inflammatory effects than beta-carotene.	[126]
Human patients						
Beta-carotene	124 women with CIN2 and 3 lesions (mean age 29.8 y.o.)	Phase III trial using oral beta-carotene supplementation.	30 mg daily	24 months	The 24-month study showed no benefit or harm for women with CIN2 or 3 treated with placebo or beta-carotene.	[127]
Beta-carotene	117 women with abnormal cervical morphology (mean age 30.1 y.o.)	Randomized, double-blind, placebo-controlled trial using beta-carotene and lecithin supplementation.	30 mg daily	12 months	No differences were observed between the beta-carotene and placebo groups regarding Pap smear results and HPV positivity.	[128]
Vitamin A	30 women with advanced cervical carcinoma (mean age 55.8 y.o.)	Randomized, double-blind, clinical trial regarding the effects of vitamin A, in advanced cervical carcinoma treated with neoadjuvant chemotherapy.	80,000 UI/8 h	64 weeks	Adding vitamin A to neoadjuvant chemotherapy treatment may improve the clinical response in advanced cervical carcinoma, but without statistically significant results.	[129]
Folate	60 overweight/obese women with CIN2/3	Randomized, double-blind, placebo-controlled clinical trial regarding the effect of folate supplementation recurrence and metabolic status of CIN2/3.	5 mg/day	12 weeks	Non-significant decrease in CIN2/3 recurrence in the folate group. The supplementation with folate significantly reduced the levels of homocysteine, insulin, and C-reactive protein, improving insulin sensitivity and antioxidant capacity.	[130]
Folate	48 women diagnosed with CIN1 (mean age 37.9 y.o.)	Randomized, double-blind clinical trial regarding the effect of long-term folate supplementation on metabolic status and regression of CIN.	5 mg/day	6 months	Folate supplementation promoted regression of CIN lesions and had beneficial effects on plasma levels of homocysteine, serum insulin, GSH, and MDA.	[131]
Folate	235 women with CIN1 and 2 (mean age 25.0 y.o.)	Clinical interventional trial regarding the effect of folate supplementation on CIN.	10 mg	6 months	There were no significant differences between the supplemented and non-supplemented subjects regarding dysplasia, biopsy, or HPV type 16 infection.	[132]
Antioxidant mixture: beta-carotene, vitamin C, vitamin E, selenium, zinc, *Ginkgo biloba* extract, and *Panax ginseng* extract	103 women with IIB and IIIB cervical cancer (mean age 48.4 y.o.)	Single-blinded, randomized clinical trial regarding the effect of an antioxidant mixture on the recurrence of cancer.	1 capsule daily	4 years after completion of antineoplastic treatment	Antioxidant supplementation had no benefit in patients with cervical cancer.	[133]
Antioxidant mixture: beta-carotene, vitamin C, vitamin E, and selenium	103 women with IB2–IIIB cervical cancer receiving chemotherapy and radiotherapy (mean age 45 y.o.)	Randomized, single-blinded, controlled trial in women with cervical cancer regarding the effect of antioxidant supplementation on oxidative stress and hematological toxicity during oncology.	1 capsule daily	6 weeks during cervical cancer treatment	The supplementation with antioxidants reduced oxidative stress and maintained hemoglobin levels in cervical cancer patients treated with chemotherapy and radiotherapy.	[134]
Vitamin D	58 women with CIN1 (mean age 37.2 y.o.)	Randomized, double-blind, placebo-controlled trial regarding the effect of long-term vitamin D supplementation on regression and metabolic status of CIN.	50,000 IU vitamin D3 daily	2 weeks/month for 6 months	Supplementation with vitamin D3 for 6 months promoted the regression of CIN1 and improved glucose parameters, as well as levels of NO and MDA.	[15]
Vitamin D3	58 women diagnosed with CIN2/3 (mean age 40.08 y.o.)	Randomized, double-blind, placebo-controlled trial regarding the effect of long-term supplementation of vitamin D3 on recurrence and metabolic status of CIN2/3.	50,000 UI vitamin D3 daily	2 weeks/month for 6 months	Supplementing women with vitamin D3 for 6 months reduced the risk of recurrence of CIN1/2/3 and improved their metabolic status.	[135]
*Coriolus versicolor*–based vaginal gel	91 HPV-positive women with CIN1 (mean age 40.5 y.o.)	Multicenter, open-label, randomized, parallel-group, watchful waiting approach-controlled trial.	Vaginal gel once daily	21-day treatment/7-day rest period, for 3 to 6 months, followed by 5 or 3 months of alternate use	After 6 months of treatment, HPV clearance was observed in 59.6% of the patients. More efficient cervical re-epithelialization was seen after vaginal gel use.	[46]
*Coriolus versicolor*–based vaginal gel	41 HPV-positive women with CIN1 (mean age 47.71 y.o.)	Multicenter, randomized, open-label, parallel-group, controlled clinical trial.	Vaginal gel once daily	21-day treat-ment/7-day rest period, for 1 to 3 months, followed by 5 or 3 months of alternate use	After 6 months of treatment, HPV clearance and lesions repair were consistent in women older than 40 years, in both high-risk HPV strains infected patients as well as in 16-18-31 HPV subtypes.	[47]
*Coriolus versicolor*–based vaginal gel	263 HPV-positive women with CIN1 (mean age 38.7 y.o.)	Observational, national, multicentric, prospective, non-comparative clinical study.	Vaginal gel once daily	21 days during the first month, then alternate days for 5 months	After 6 months of treatment, higher regression rates of low-grade cervical lesions were seen in women who received the treatment.	[48]

CIN—cervical intraepithelial neoplasia; GSH—glutathione; HPV—Human Papillomavirus; MDA—malondialdehyde; NO—nitric oxide.

Known for its ability to eliminate ROS and RNS, curcumin is one of the primary compounds found in turmeric rhizomes. This compound interacts with free radicals by electron transfer, followed by proton loss or direct hydrogen atom abstraction, primarily due to the phenolic OH group [82]. Activation of NF-κB has been associated with all stages of carcinogenesis, and studies have shown that curcumin inhibits its activation, thereby benefiting through induction of cell death, prevention of tumor growth, and blocking metastatic progression [136]. Moreover, curcumin can influence the regulation of cell proliferation, apoptosis, angiogenesis, cancer-associated inflammation, and drug resistance by directly or indirectly binding to specific molecular targets. This compound has also been investigated in combination with other natural or synthetic compounds to overcome the limitations of chemotherapy and reduce side effects [137]. In a recent study, Purba et al. [121] examined the effect of curcumin on HeLa cells, focusing on the regulation of p53 and caspase-3 protein expression. Curcumin concentrations used ranged from 25 to 250 µg/mL, and treatments lasted for 24 h. The results showed that curcumin treatment significantly increased apoptosis and upregulated p53 and caspase-3 protein expression [121]. Shang et al. [138] confirmed that curcumin induced cell death in HeLa cervical cancer cells by damaging DNA and chromatin condensation.

Predominantly found in a wide range of medicinal plants, rosmarinic acid is another polyphenolic compound which is known for its ability to modulate the activity of enzymes involved in OS, including CAT, SOD, GPx. The ability to modulate these antioxidant enzymes makes this acid a powerful agent to protect cells against oxidative damage, especially in cancer, where OS plays a key role in disease progression [139]. Furthermore, rosmarinic acid at concentrations of 100–1000 µg/mL for 48 h showed limited efficacy on HeLa cells, suggesting its reduced effectiveness compared to other types of cancer [120].

The antioxidant 6-Gingerol also has various biological effects, including anticancer properties [140,141]. It was found that 6-gingerol at a concentration of 0–50 μmol/L, administered for 24 h, also inhibited HeLa cell invasion and migration, as shown by Xing-Yu et al. [123]. Finally, kaempferol is another flavonoid widely distributed in a variety of medicinal plants that has demonstrated anticancer activity. It was demonstrated that at a concentration of 30–50 μM for 48 h, it induced apoptosis and cell arrest in the G2-M phase, highlighting its potential as an anticancer agent [119].

Vitamin A is an antioxidant responsible for healthy development, maintenance of epithelial tissues, and its ability to inhibit early events in cervical carcinogenesis [12,142]. Deficiency of this vitamin exposes the patient to additional effects of OS, inhibiting any cellular repair. In one study, Srivastava et al. [142] reported that vitamin A levels decreased significantly across different stages of cervical cancer, from stage I to stage IV. Vitamin C is a potent antioxidant that can enhance the effect of chemotherapeutic agents without increasing their toxicity in normal cells [82]. Hwang et al. [143] demonstrated that women with high-risk HPV could benefit from dietary supplementation with vitamin C, as it could reduce the risk of CIN. It has been shown that women with CIN have lower plasma levels of α-tocopherol (vitamin E) compared to normal subjects [82]. Furthermore, it has been found that patients with cervical cancer had lower serum levels of vitamin E [140]. In a hospital case-control study, Guo et al. [144] demonstrated that higher serum concentrations of α-carotene, β-carotene, Vitamin E, and vitamin C were associated with a lower risk of cervical cancer in women. This highlights the importance of antioxidant vitamins in the process of carcinogenesis and encourages the consumption of fresh vegetables and fruits rich in antioxidant enzymes [144].

Most of the studies regarding the antitumoral and antioxidant effects of natural compounds are generally performed on HeLa cell cultures, and the more advanced trials on human patients. However, several animal model studies have been reported regarding this aspect (Table 3). In this way, Palanisamy et al. [124] reported the antioxidant and chemotherapeutic effect of *Curcuma amada* extract in benzo(α)pyrene-induced cervical carcinoma in 30 female Sprague Dawley rats. Oral administration of the extract resulted in a reduction in tumor burden and restoration of antioxidant levels, as well as a decrease in lipid peroxidation [124]. Also, treatment with vitamin E on cervical epithelial carcinogenesis in female Swiss albino mice resulted in a reduction in the incidence of dysplasia and prevention of carcinoma in situ, improving antioxidant enzyme activity and reducing lipid peroxidation [125]. In another study, protection provided by curcumin and beta-carotene against cisplatin-induced ovarian damage in female Wistar albino mice was demonstrated [126].

On the other hand, many observational studies have shown an inverse relationship between the supplementation with antioxidants and the risk of developing preinvasive neoplasia or cancer, including cervical cancer [15,127,128,129,130,131,132,133,134,135] (Table 3). In their study, Keefe et al. [127] evaluated the effect of 30 mg of β-carotene over a period of 24 months in women with CIN 2 and 3. The study results confirmed that β-carotene did not improve the regression of CIN 2 and 3, especially in patients who tested positive for HPV [127]. On the other hand, daily administration of 30 mg of β-carotene over 12 months had no significant effects on cervical cytology or the amount of HPV DNA in the genital samples of women with abnormal cervical morphology [128]. Sanusi [129] found a greater reduction in tumor size in the neoadjuvant chemotherapy (cisplatin and paclitaxel) + vitamin A group compared to the neoadjuvant chemotherapy-only group. These results suggest that vitamin A supplementation influences and mediates changes in the tumor volume of cervical carcinomas [129].

Folate supplementation did not have a significant impact on the recurrence of CIN 2/3 but significantly reduced homocysteine, insulin, and C-reactive protein levels, improving insulin sensitivity and antioxidant capacity [129]. On the other hand, Asemi et al. [131] evaluated the effects of folate supplementation on regression and metabolic status in patients with CIN 1. The study results confirmed that daily folate supplementation for 6 months in women with CIN 1 led to its regression [131]. In the study by Butterworth et al. [132], folic acid supplementation did not affect the progression of cervical dysplasia or HPV-16 infection, although folate deficiency may contribute to the initiation of dysplasia.

In another study, Alvarez-Altamirano et al. [133] conducted a controlled trial on cervical cancer patients receiving 25 sessions of radiotherapy (50 Gy) and cisplatin chemotherapy (40 mg/m^2^), supplemented with antioxidants or placebo for four years, evaluating the effect on relapse. The antioxidant supplement administered to the antioxidant group contained β-carotene (30%), vitamin C, vitamin E, selenium yeast, zinc oxide, Ginkgo biloba extract, and Panax ginseng extract. The results showed that antioxidant supplementation did not offer any benefits [133]. In a single-blinded, randomized, controlled trial, antioxidant supplementation reduced oxidative stress, maintained hemoglobin levels, and improved the quality of life in the antioxidant group. However, further studies are needed to evaluate long-term effects of these mixtures [134].

A randomized, placebo-controlled study conducted by Vahedpoor et al. [135] confirmed that supplementation with vitamin D3 in women with CIN 1 for 6 months promoted its regression and had beneficial effects on glucose parameters, plasma NO levels, total antioxidant capacity, total glutathione, and MDA. Beneficial effects were also observed on insulin metabolism markers [15].

## 5. Materials and Methods

The current narrative review was based on a thorough search on the most known scientific databases (Web of Science, PubMed, ScienceDirect, Scopus, Google Scholar, Cochrane Systematic Reviews Library) and was performed using keywords, such as “cervical cancer”, “cervical intraepithelial neoplasia”, “HPV”, “oxidative stress”, “inflammation”. The scientific articles were screened based on publication date (from January 1990 to date), language (English), title, abstract, availability (full text), and scientific relevance and soundness. The description of the selected studies was performed by narration in the text and summarization in the tables.

## 6. Conclusions and Future Perspectives

Cervical cancer is a significant health concern that develops progressively through stages of CIN, with chronic HPV infection identified as the primary etiological factor. The progression from CIN to invasive cervical cancer is influenced by multiple factors, including the immune system’s ability to control the virus and the oxidative stress. While lower-grade lesions (CIN1) may regress spontaneously, higher-grade lesions (CIN2 and CIN3) are associated with a higher risk of progression to cancer, often necessitating medical intervention. Timely detection through routine screening and appropriate therapeutic measures remains crucial in preventing the development of cervical cancer.

**Figure 1:**
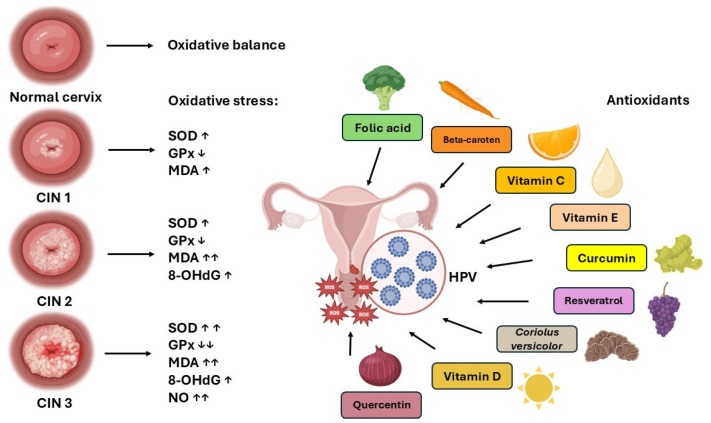
Oxidative stress markers and antioxidants in CIN (image partially generated in BioRender). ↓—significant decrease; ↑—significant increase.

Oxidative stress and chronic inflammation are central to the pathophysiology of CIN, promoting DNA damage, immune evasion, and cellular mutations. Antioxidants play a pivotal role in mitigating the oxidative stress associated with cervical cancer. SOD, CAT, and GPx neutralize ROS, while GST and GR contribute to detoxification processes. Non-enzymatic antioxidants, including GSH and vitamins C, E, and A, support cellular repair mechanisms and detoxification. Phytochemicals like quercetin, found in tea, nuts, and vegetables, have been shown to inhibit HeLa cell proliferation, induce apoptosis, and enhance the efficacy of chemotherapy. Resveratrol, a polyphenol present in grapes and wine, suppresses cancer cell growth by activating FOXO3a and inhibiting the oncoproteins E6 and E7 of HPV. Curcumin, the principal compound in turmeric, regulates NF-κB signaling, induces apoptosis, and hinders tumor progression. Other bioactive compounds, such as rosmarinic acid, 6-gingerol, and kaempferol, exhibit promising anticancer effects by inducing cell cycle arrest and apoptosis (Figure 1). Additionally, vitamins A, C, E, D, and folate play a role in antioxidant defense, with some studies suggesting potential benefits in reducing the progression of CIN, though clinical evidence remains limited. A deeper understanding of these antioxidant mechanisms could enhance strategies for improving cervical cancer prevention and treatment outcomes.

In conclusion, incorporating antioxidant-rich strategies alongside regular screenings and timely medical interventions could significantly improve the prevention, management, and overall prognosis of cervical cancer, offering hope for more effective treatment options and better patient outcomes.

## Figures and Tables

**Table 1:** Stabilization of CIN.

Stages of CIN	Description
CIN1	Low-grade lesions caused by active and transient HPV infection. The lesions are mild dysplastic and could be found in the lower third of the cervical epithelium [42].
CIN2	Moderate-grade lesions characterized by increased cellular atypia and mitotic activity. The lesions extend to two-thirds of the epithelium thickness [43].
CIN3	Severe-grade lesions are characterized by severe dysplasia with high mitotic activity and loss of normal epithelial stratification. The lesions extend to the entire epithelial thickness. Similar to CIN2, the CIN3 lesions predispose to malignant processes [40].

**Table 2:** Enzymatic antioxidant activity in patients with CIN and cervical cancer.

Study	Design	Cervical Cancer Description	Samples	Results	Observations	Ref.
Investigation of circulating LP and antioxidants in cervical cancer patients.	Case-control study: 30 CIN patients, 44.2 ± 8.0 y.o., non-smokers, 30 healthy controls, 44.3 ± 8.38 y.o., non-smokers	Clinical classification: stages II and III	Hemol-ysate	SOD ↓ GPx ↓ GST ↓ GSH ↓	The neutralization of lipid peroxides and sequestration by tumor cells could be the primary causes of antioxidant level decrease.	[85]
			Plasma	Vitamin C ↓ Vitamin E ↓		
Evaluating the level of oxidative stress in cervical cancer patients and the age-matched healthy controls.	Case-control institutional study: 100 patients, 28 healthy controls, 34.46 ± 5.82 y.o.	Clinical classification: FIGO stages I, II, III, and IV	Serum	MDA ↑ SOD ↑ GSH ↓ 8-OHdG ↑	This imbalance between antioxidants and oxidants in cervical cancer significantly contributes to the pathogenesis and progression of the disease, being more pronounced in advanced stages.	[9]
Comparison of antioxidant status in cervical cancer patients vs. healthy controls.	Case-control study: 35 patients, 35 healthy controls, 38–79 y.o.	FIGO I, II, III	Hemol-ysate	GPx ↓ SOD ↑	Antioxidant status is altered in cervical cancer patients, but its link to carcinogenesis is unclear. GSH may predict treatment response.	[86]
			Plasma	MDA ~		
To assess antioxidant levels and biomarkers of oxidative stress in cervical cancer and compare these levels between patients and healthy women.	Case-control study: 120 patients with cervical cancer, 30 healthy controls, 25–65 y.o.	Cervical cancer stages: I, II, III, and IV	Serum	MDA ↑ NO ↑	Increased MDA and NO indicate DNA damage. Vitamin C hypovitaminosis exacerbates the damaging processes, suggesting a significant negative impact on cellular health.	[87]
			Plasma	Vitamin C ↓ RBC-SOD ↓		
Observational pre- and post-treatment analysis of antioxidant levels in cervical cancer patients.	Case-control study: 30 patients, 48.8 ± 11.4 y.o., 30 heathy controls, 49.0 ± 13.4 y.o.	Cervical cancer stages: I, II, III	Plasma	GSH ↓	Cervical cancer patients showed lower plasma and erythrocyte glutathione, and glutathione peroxidase activity compared to controls, indicating a weakened antioxidant defense system.	[88]
			Hemol-ysate	GSH ↓ GPx ↓		

GPx—glutathione peroxidase; GSH—glutathione; GST—glutathione S-transferase; LP—lipid peroxidation; MDA—malondialdehyde; NO—nitric oxide; RBC-SOD—erythrocyte superoxide dismutase; 8-OHdG—8-hydroxy-2′-deoxyguanosine; ↓—significant decrease; ↑—significant increase; ~—no significant change.
